# Perspectives of Patients, Doctors and Medical Students at a Public University Hospital in Rio de Janeiro Regarding Tuberculosis and Therapeutic Adherence

**DOI:** 10.1371/journal.pone.0137572

**Published:** 2015-09-11

**Authors:** Elizabeth da Trindade de Andrade, Élida Azevedo Hennington, Hélio Ribeiro de Siqueira, Valeria Cavalcanti Rolla, Celina Mannarino

**Affiliations:** 1 National Institute of Infectious Diseases Evandro Chagas (INI/Fiocruz), Rio de Janeiro, Brazil; 2 Deputy Direction of Education, National Institute of Infectious Diseases Evandro Chagas (INI/Fiocruz), Rio de Janeiro, Brazil; 3 Faculty of Medical Sciences, State University of Rio de Janeiro (UERJ), Rio de Janeiro, Brazil; 4 Clinical Research Laboratory on Mycobacteria, National Institute of Infectious Diseases Evandro Chagas (INI/Fiocruz), Rio de Janeiro, Brazil; California Northstate University College of Medicine, UNITED STATES

## Abstract

**Introduction:**

The World Health Organization (WHO) identifies 8.7 million new cases of tuberculosis (TB) annually around the world. The unfavorable outcomes of TB treatment prevent the achievement of the WHO’s cure target.

**Goal:**

To evaluate existing intersections in the conceptions relative to the knowledge of TB, the experience of the illness and the treatment.

**Methods:**

Doctors, medical students and patients were selected from a public university in Rio de Janeiro, Brazil, from 2011 to 2013. The data were obtained by semi-structured individual and focus group interviews, participant observation and a field journal. The inclusion of patients was interrupted due to saturation, and the inclusion of doctors and medical students stopped due to exhaustion. The theoretical background included symbolic Interactionism, and the analysis used rounded Theory. The analysis prioritized the actions/interactions axis.

**Results:**

Twenty-three patients with pulmonary TB, seven doctors and 15 medical students were included. In the interviews, themes such as stigma, self-segregation, and difficulties in assistance emerged, in addition to defense mechanisms such as denial, rationalization, isolation and other mental mechanisms, including guilt, accountability and concealment of the disease. Aspects related to the assistance strategy, the social support network, bonding with the healthcare staff and the doctor-patient relationship were highlighted as adherence enablers. Doctors and students recommended an expansion of the theoretical and practical instruction on TB during medical students’ education. The existence of health programs and policies was mentioned as a potential enabler of adherence.

**Conclusion:**

The main concepts identified were the stigma, self-segregation, guilt, responsibility, concealment and emotional repercussions. In relation to the facilitation of therapeutic adherence, the concepts identified were the bonds with healthcare staff, the doctor-patient relationship, assistance and educational health strategies.

## Introduction

TB is a contagious disease that is transmitted through the respiratory tract, with significant incidence in urban areas, especially in developing countries. The disease is associated with poor housing and feeding conditions, lack of sanitation, alcohol abuse, tobacco and other drugs, and the HIV/AIDS epidemic [1].

Approximately 8.7 million new cases of TB are identified annually throughout the world, and 1.7 million deaths occur every year, making TB the primary cause of death by curable infectious disease [2].

Brazil is in 17^th^ place among the 22 countries with substantial TB burdens in the world. In 2012, there were 70,047 new cases, with approximately 4,600 deaths [3]. The percentage of individuals successfully treated and cured was approximately 70%, indicating that the country is far from the target established by the WHO (85% of patients cured) [4]. Approximately 10% of patients (in Brazil) do not adhere to treatment, which is another important obstacle for TB control that contributed to the increase in multi-resistant TB cases between 2001 and 2010 [4–5].

The State of Rio de Janeiro, with its social contrasts, has the highest incidence of the disease in the country (72.3/100,000 in 2011, with the emergence of 11,651 new cases). Of these cases, approximately 80% were pulmonary and 20% were extrapulmonary TB. The mortality rate in 2010 was 5.6%, and the proportion of patients who did not adhere to treatment was 11.5% in 2011 [5–6]. The long therapeutic regimens (six months) and improvement of symptoms after the second month of treatment favors non-adherence as well as the occurrence of adverse effects [7]. This scenario, associated with the historical representation of TB, which includes stigma, death, high transmissibility, lack of knowledge about the disease and difficulties in assistance, indicates the urgent need to create adherence strategies to interrupt the disease transmission cycle and improve the cure rate.

Previous qualitative studies on adherence to TB treatment prioritize the inclusion of patients and sometimes include healthcare professionals or students. However, these studies have not explored the uniqueness of the emotional and social repercussions resulting from pulmonary TB diagnosis (and treatment) [8,9,10].

The aim of this study was to evaluate existing intersections in the conceptions relative to the knowledge of TB, the experience of the illness and the treatment, as they influence treatment adherence and the relationships of patients, doctors and medical students.

## Method

The study included patients, doctors and fourth-year medical students. Patients were adults of both genders in their first episode of pulmonary TB who attended the TB ambulatory clinic of a public university in the state of Rio de Janeiro, Brazil, from November 2011 to December 2013. The study excluded patients being re-treated for TB; those with HIV/AIDS, neoplasia, or other infectious diseases; transplanted patients; pregnant women; nursing mothers; visually, physically or hearing impaired patients; and medical students who had TB in the past. No exclusion criteria were used for doctors.

Preceptor doctors and fourth-year medical students were invited to participate in the study if they worked with pneumology outclinic patients.

The sample of patients was defined by the theoretical saturation criterion [11], in which the interruption of the inclusion of new participants occurred when the data obtained began to present redundancy or repetition. For doctors and medical students, the sample was defined by exhaustion [12]. The most important characteristic of the sample of patients, doctors and medical students, was the inclusion of subjects directly involved with the illness and the tuberculosis treatment.

The participants were selected gradually according to consultation attendance (for patients with their first diagnosis of TB) or during their activities in the ambulatory (for physicians and medical students in training and education in the clinic). The doctors knew about the interest of the interviewer, a female master’s student in investigating the process of experiencing patients with TB and their treatment. The students and patients did not know the interviewer; they met the interviewer face to face at the time of selection.

Semi-structured individual interviews were used with all participants (medical students, doctors and patients), with an average duration of 50 minutes, and they were conducted by the same interviewer. The interviews were conducted in a private room in the out clinic space; only the participants and the researcher were present.

The interviews were conducted based on a screenplay previously prepared by the authors focusing on the topics of interest. (Table 1). No closed questions were used. The questions were easy to understand and presented in everyday language. The flexibility in the conduct of the interviews allowed for reorientation and adjustment when necessary. Hence, there was no need to repeat the interviews. A pilot test was not conducted.

**Table 1:** Guide for individual interviews with patients, preceptor doctors and medical students.

Interviewed	Questions
Individual interviews with patients	What does tuberculosis mens to you?
	How did it feel to find out for you had tuberculosis?
	Talk about the treatment of tuberculosis that was explained to you.
Individual interview with the patients (after the completed treatment)	How was the experience of TB illness and treatment?
Individual interviews with preceptor doctors	What do you think the patient knows about TB?
	How do you perceive the meaning of the diagnosis to the patient?
	What does it mean to you to give the diagnosis of TB to the patient?
	What does adherence to TB treatment mean to you?
	What can affect the patient’s compliance with TB treatment?
	What does TB mean to you?
Individual interview with the medical students	What do you know about TB?
	What do you think the patient knows about TB?
	How do you perceive the meaning of the diagnosis to the patient?
	How do you interpret adherence to TB treatment?
	What do you think can interfere with the patient’s compliance with the treatment of pulmonary TB?

The individual interviews with the patients were conducted at the beginning of treatment to determine the emotional repercussions of the diagnosis and their knowledge about TB as well as at the discharge consultation to understand the patients’ experience of the treatment process.

The individual interviews with the preceptor doctors were designed to learn about their clinical experiences in the TB approach and in the adherence to treatment and interventions that can prevent non-adherence to therapy, as well as proposals to improve medical education that can directly affect the doctor-patient relationship and, consequently, patients’ adherence.

The patients were part of focus groups after two months of treatment. The focus groups lasted an hour and a half on average, and the discussion strategy included developing the history of a hypothetical patient who received the TB diagnosis, a report of their experiences related to the experience of falling ill and implications of adherence to TB treatment. In the students’ focus group, the factors involved in the patients’ therapeutic adherence were discussed. The following question was asked: "What are the factors involved in adherence to TB treatment?”

Individual and group interviews were audio recorded and transcribed by the interviewer (a psychologist and master’s student), who had individual and group clinical experience and who discussed the use of interviews with the thesis advisors. The data and information were collected and analyzed by the group of researchers without the use of software. Quotations from the participants are used to illustrate the issues: the patients (P) and physicians (Dr.) were identified by numbers, and the students (S) were identified by letters, aiming to ensure their anonymity.

The analysis included the technique of triangulating various sources of reports [13–14]: those from patients, doctors and medical students and obtained from interviews, participant observations and a field journal. This technique is useful in qualitative research, as using more sources of information can assist in better understanding a phenomenon. Field notes were made after the interviews were concluded. Throughout the study, a field journal was used, and participant observations were made.

We adopted the theory of symbolic interactionism, which states that human beings attribute individual and collective actions to their experiences and consequences [15], denoting the social, assistance and educational spheres of health in this study.

As a variant of symbolic interactionism, grounded theory (GT) was used for the analysis, with the aim of understanding phenomena or reproducing them according to the comprehension of the individual and to obtain a theoretical construction through systematic data analysis to explain the action in a social context [16,17,18,19]. GT analyzes statements in three non-sequential phases: open codification—attainment of concepts and categories; axial codification—relationship of the categories and subcategories; and selective codification—organization of the findings and the establishment of a central category and/or a new theory [17,18,20].

The axial codification organizes the categories according to a phenomenon based on the following conditions: *Causal* (a set of facts or events), *Intervenient* (unexpected facts that affect the impact of causal conditions), *Context* (the reason for the phenomenon—circumstances and situations), *Actions/interactions* (strategic responses in situations, problems and issues among people, groups and institutions), and *Consequences* (the results of actions or their failures) [17].

This article discussed the categories that emerged from the process of interpretative analysis in the “actions/interactions" axis for expressing intersubjectivity in the participants’ relationship and the experience of illness and TB treatment, thereby covering the social, personal and institutional contexts.

We emphasize concepts contributing to the intersection between participants (in dyad or triads); however, to enhance the understanding of these phenomena and to consider the relevance of other content belonging to the actions/interactions axis, we also included concepts that emerged only in student and patient samples.

This work was approved by the Research Ethics Committee of the National Institute of Infectious Diseases Evandro Chagas (INI/Fiocruz) under the CAAE number 08550112.4.0000.5262 and of the public university in the state of Rio de Janeiro, Brazil. All participants voluntarily signed the Informed Consent Form.

## Results and Discussion

The characteristics of TB are based on the political, social, economic and scientific contexts throughout its history that emotionally compromise patients and those who participate in their care, including doctors and medical students. Based on the different meanings attributed to the events and experiences of individuals, according to symbolic interactionism, we sought to understand the subjective aspects that emerged from the TB diagnosis for patients and to relate them to concepts of therapeutic adherence based on the perceptions of fourth-year medical students and the doctors who cared for these patients.

Twenty-three patients, seven doctors (all medical preceptors who attended the TB clinic) and 15 students were recruited and informed about the purpose of the study. None of the recruited subjects refused to participate, and none withdrew during the study. Although the data have not been discussed with the participants, they can be in the future after the final publication of this article. Simple language will be use to explain the results to patients.

The following categories that expressed intersections on the "actions/interactions" axis were identified in the interviews with doctors, patients and medical students: stigma, self-segregation, emotional repercussions, guilt, accountability, concealment, bonding with the healthcare staff, the doctor-patient relationship and care strategies.

“Difficulty in health care” was a category identified only by doctors and patients, the “social support network” category was a theme expressed by students and patients, and “TB and society” was mentioned by doctors and students. The “medical obedience” and “difficulties of students” categories emerged only in the discussions with students. The same is true for the "denial" category, which is a mental defense mechanism only applying to patients. Although these categories do not represent intersections of concepts among these three or two groups of participants, they were considered relevant in the context.

The main themes were identified in advance, but new themes were derived from the data. The themes addressed in the interviewees were divided into the following subthemes to aid in comprehension in the analysis: “understanding TB”, “understanding theexperience of falling ill with TB”, and “training future doctors”.

### Understanding TB

The category **stigma**, among the several meanings related to the illness, treatment and cure of TB, was a distinct social concept associated with the disease, revealing the social reality of the subjects.

According to the students and doctors, consideration of this **stigma** may be a way to improve adherence (because of the fear that patients will always have this condition) or cause non-adherence (because of the negative influence of the conception of TB in the social context of patients, if segregation and discrimination prevent health care delivery). This fear expressed by patients is highlighted in the following statement by a student:

*I kind of think that it’s… it’s something kind of from the past*, *because in the past*, *the person who had tuberculosis was an evening person… “bon vivant” and all… who was out there “bumming around a lot”*. *So*, *I think this stigma*, *some people still carry it*. *(…) … Kind of like “if I have tuberculosis*, *either I’m going to be isolated… people will look at me in a weird way… they will jump to conclusions”*.

*(S—M)*

The historical traces of the **stigma** of TB embedded in the institutional norms and in communities extend beyond social and economic issues. Many studies have identified the stigma surrounding TB in developing countries by associating it with poverty, poor housing conditions and cultural variations with regard to conceptions and behaviors [21,22,23] as well as with delays in the search for assistance, often reflected in delayed TB detection [24]. However, a recent study conducted in Denmark, a country with high incomes and low TB prevalence, reports that this stigma is experienced by Danish patients on a similar level and leads to harm in social interactions and delayed diagnosis [25]. Regardless of the economic situation of the country or social class, the stigma surrounding TB exists, and it hinders diagnosis and increases the likelihood of non-adherence to treatment, thus reinforcing the morbid identity of individuals as reflected in their actions and interactions in social contexts.

The fear of experiencing the stigma and transmitting the disease, especially to their family members, was mentioned by the patients as an enabler of **self-segregation** (personal isolation); this behavior was observed by the doctors, and it causes patients to avoid health care and consequently abandon it. This behavior was also highlighted in a study of the psychosocial experiences of patients with TB, signaling that medical leave from work can also be a resource of self-segregation, not only because of health conditions but also because of the desire to avoid social and emotional embarrassment [21]. Another study of nurses reinforced the idea of self-segregation as a strategy to escape this stigma [26].

The following quotation of a medical student expresses this type of thinking:

*It can contaminate other people… There’s also that… social part*, *where the person sometimes does not want to contaminate other people*, *and people stop making contact…*

*(S—K)*

The **emotional repercussions** (feelings resulting from the diagnosis, illness and treatment process) permeated the statements analyzed in this study. Similar to the patients when they received the TB diagnosis, the doctors also experienced emotional effects of giving the diagnosis as a result of the significant interactions established throughout treatment. The six-month treatment course brings doctors, patients, and family members closer, tightening these empathetic bonds. A doctor reported that TB is associated with the following ideas and factors: a frightening and serious disease, a lack of knowledge of transmission routes and treatment; the idea of death; being surrounded by fear, concern, sadness and prejudgment; and an emotional shock. All the doctors observed the emotional impact of the TB diagnosis on their patients. However, these consequences were not assessed. Only one doctor emphasized the importance of treatment adherence:

*If this sick person does not suffer from the impact of the diagnosis*, *they won’t… they won’t treat themselves correctly*. *Because it’s the impact that produces the need for the sick person to take the medication correctly*. *If there’s no impact*, *then the person doesn’t care what they have and will probably abandon the treatment*. *I mean… it’s somewhat a paradox*. *The impact is unpleasant*, *but at the same time*, *we know that the sick person having an impact*, *they will… they will adhere to the treatment and be cured*

*(Dr*. *1)*.

Considering the empathetic relationship, another doctor explained as follows:

*The diagnosis is scary for the patient*. *Because… the idea people have is that tuberculosis is a disease that kills a lot and*, *(…) from the patient’s point of view*, *there is a strong impact because of that*.

*(Dr*. *4)*

According to the doctors, the meaning of TB involves a curable disease, and they defined it as exemplifying an unequal society. For some doctors, TB also represented a challenge:

*(…) Because it involves the social*, *(…) political (…) economic issue*, *the country as a whole*, *because it is a neglected disease*, *(…) from poverty*. *On the one hand*, *we technically have how to act*, *the right thing and all*, *and on the other hand*, *there is a disease that*, *comparatively*, *there’s no control*, *despite all this*. *It’s a challenge*.

*(Dr*. *4)*

The strategic actions taken by the doctors to mitigate the impact of the diagnosis and to make treatment adherence easier included the following: exercising prudence in giving the diagnosis, emphasizing the possibility of a cure, recommending the use of masks at home, and identifying contacts through exams and attendance at the latent TB infection clinic.

Among the patients, the physical and emotional impacts of the experience of the illness, relevant factors and reasons for concern regarding treatment adherence were strongly expressed because the debilitating symptoms create feelings of inability, impotence and self-discrimination [21]. The patients revealed experiences of rejection, discrimination, fear, concern, distrust in the diagnosis, aversion to medication, apprehension, nervousness, guilt, inability, surprise, sadness, desperation and fear of being contagious. One patient questioned her ability to complete the treatment:

*I don’t really know how to get there with this because to me*, *it was a surprise and a strike in my personal life*.

*(P—5)*

Consequently, behaviors related to self-segregation, concealment of the disease and sleep disorders emerged, interfering with the course of treatment.

The medical students showed difficulty in understanding the difference between the information offered by the doctor and the comprehension, feelings and behaviors of the patients related to their disease and treatment. The students revealed **emotional repercussions** as a *consequence* of their identification with patients while performing their role as doctors.

*It is explained*, *the doctors explain*, *but I don’t know why they don’t… they don’t adhere*. *Yeah*. *They abandon the treatment*. *A bacillus that*, *if it gains resistance*, *is so dangerous*. *If it’s resistant to antibiotics*, *there is almost no option for treatment*. *It’s a very serious disease*. *I don’t know*.

*(S—I)*

The patients felt excited and relieved at the end of the treatment because they had addressed the adverse effects and because they could no longer transmit TB. Although they were informed that they were no longer contagious after 15 days of medication, the patients did not believe that they were no longer contagious until they completed the treatment.

*It was great*! *… Because you get out of the six months that you’ve spent not doing anything… then you go back to work*. *Then you go back to work full force… With high spirits and all…*

*(P—16)*

### Understanding the experience of falling ill with TB

Several notions observed in the individual interviews also emerged from the interactions of the participants in the groups of patients and students. In the focus groups with patients, we categorized the explained content as **Group/Interaction/Adherence** (interaction facilitates adherence), and in the focus group of students, we categorized the content as **Group/Interaction/Meaning** (interaction facilitates reflection). This methodological group strategy has been applied in several qualitative studies in various countries and as a health and therapeutic adherence educational device [21,27,28,29,30].

The following statement by a patient in the group regarding the gains that can be associated with the disease contributed to the self-esteem of another patient, who emphasized the negative points in his life resulting from the TB:

*And sometimes we think the disease is coming to bring us down… to take us*, *right*, *let’s say*, *kill us*. *And we learn so much with it*. *…that it comes only as a mechanism for us to think and realize what we need to change*. *To improve our health even more*, *got it*? *Because you’re treating tuberculosis*, *but look at what you won with it*. *You’re freeing yourself from the cigarette addiction*, *the alcohol addiction… Look how great that is*. *Your life will be completely different… your diabetes will become more controlled… think about it*.

*(P—15)*

The positive reinforcement among the patients revealed attitudes of encouragement and tranquility with regard to the meanings attributed to their experiences, not in restricting the disease to the biological aspects.

*Yeah*, *but we can’t keep thinking that it will come back*. *Otherwise it comes back*. *(…) You have to forget about it*, *too*. *Everything is in the psychological side of the person*, *you know*. *If we think the cough will come*, *you will be coughing later*.

*(P—4)*

The group interaction enabled patients to discuss their intention to abandon treatment:

*Because*, *I mean*, *I listen to people saying “why are you feeling sick… it’s affecting your liver and all; this is a problem in your liver and whatever”*. *And we get scared*, *you know*? *With time*. *Yeah… That’s my fear*, *you know*? *Sometimes I even wanted to stop taking the medication…*

*(P—3)*

The attitude of abandoning treatment showed that the patient needed assistance in their social network, leading to **guilt and accountability** with regard to the non-adherence behavior. However, in their statements, the doctors and the students showed that they valued the culture, personality and bonds of patients in assessing their accountability in the treatment. This conception at times made them feel unable to meet the social demands of the patients because they became aware that therapeutic effectiveness is not restricted to medication adherence.

In the exchange of information among the students, the lack of knowledge about the free availability of medications in the Brazilian public health care system emerged, signaling the need to emphasize basic knowledge about how the Brazilian Unified Health System and its programs work during medical education, with the aim of achieving universal, contextualized, equitable and comprehensive treatment.

The **concealment** of the disease by the patients (to hide the disease because of the fear of social stigma) was a category perceived by the doctors and students as a guarantee of social coexistence. Ashamed and afraid of rejection, the patients sought to avoid negative reactions by choosing not to communicate their disease to those in their social networks. This behavior was also observed in the relationships with healthcare staff [31,32,33].

*… at the beginning… I didn’t go out a lot*. *I was very thin… People even used to think I was on drugs… I lost a lot of weight*. *Many people don’t know about this treatment that I do*.

*(P—8)*.

One student expressed the following:

*The person already expects it not to be told*. *I think it’s something like… There’s this thing about hiding the tuberculosis*. *(…) The person doesn’t tell… And that’s it*.

*(S—N)*

In the case of death by TB in the community, concealment of the diagnosis reinforced the sense of stigma and segregation. These aspects were also mentioned in the study conducted by Courtwright and Turner [23], who found that concealment is more pronounced if a TB death occurs in the family, hindering screening for the disease in the community and preventing other family members from being treated to interrupt the transmission cycle. This situation is depicted in the experience of the patient below:

*In my family… among my relatives… for a long time*, *they’ve said that… four of my relatives died from tuberculosis*. *For many years*, *they said that there were the healthcare people*, *you had to go there to take a test because they said that… the yard was contaminated with this disease ((spoken lowly))*. *Then my uncle went there with this disease… Everyone thought he was the one who brought the outbreak of the disease over there and says that the outbreak is there*. *Then everyone who dies*, *they say they died from tuberculosis*. *But really*. *Two of my uncles died*.

*(P—6)*

**Difficulties in assistance** (referring to clarification of the diagnosis and treatment) were emphasized by the patients and doctors. From the doctors’ perspective, the profile of patients with TB has changed.

*Currently*, *patients are seeking treatment earlier (…)*. *The patient arrives with condensation in their lung*, *with symptoms… That may be tuberculosis or not*, *and the sputum is positive (…) when the direct sputum is negative…*, *we need to question beyond the culture*, *which takes from 35 to 45 days…*, *also bronchoscopy and computed tomography*. *This increases the price that the hospital pays for the diagnosis*. *And (…) the possibility that we have several exams without getting the diagnosis*.

*(Dr*. *1)*

The following report of a patient shows this **difficulty in assistance** experienced as an iatrogenic act:

*With no direction*, *then… Until I came by (…) with everything right*, *with a tuberculosis report*. *I arrived at the hospital (…)*, *he said*: *“Oh*, *you don’t have tuberculosis”*. *He prescribed me I don’t know what*, *a medication and all*. *He said it was a disease that had no cure… That sooner or later I was going to lose my lung*, *that there was no way*, *etc*. *And that was the final diagnosis*. *By the lung specialist*, *who probably didn’t know anything*. *And it was this rush*, *three years without treatment*.

*(P—10)*

This statement reveals the lack of education of some specialists in the area of TB, the disregard for the diagnosis found in another institution and the breaking of the bond of trust with the healthcare professional. Some of these aspects were expressed by staff as barriers to controlling TB [34]. The patient’s lack of success in finding health care also caused feelings of hopelessness that led to delays in the diagnosis [27]. As a *consequence* of these patients’ experiences, there was a risk of clinical deterioration and the exposure of other people to the disease.

A study conducted in Burkina Faso [10] demonstrated that the price of TB medications is a primary obstacle to the continuity of treatment, as it affects the **difficulty in assistance** and financial difficulty experienced by these patients. In Brazil, the medications are free, which can help adherence and mitigate the negative influence of social, cultural, and subjective factors.

One patient noticed that the medications had no package insert, thus providing no access to information related to the treatment and adverse effects. Receiving such information is a right of the patient and entails an *intervening factor* that caused anguish and doubt with regard to continuing treatment, especially if the visits with doctors were not enlightening.

*The problems that I have now are pain*, *here*, *there and elsewhere*! *Without me knowing what it is*. *I don’t know if the medication also causes this*, *because there are no instructions for us to read to know the reactions the medication gives us*.

*(P—5)*

Some patients were encouraged to tell the doctor about their doubts and fears through a joint consultation with a psychologist. Others, however, felt comfortable asking for clarification about the treatment. The professionals aimed to be receptive to the patients in their consultations, aiming to create easier communication.

As noted by the study participants, the main reasons for a patient with TB to abandon the treatment were the following: the long duration of treatment (at least six months); difficulties in assistance; disregard for the social context and the patient’s beliefs and conceptions about health and sickness; adverse effects of medications; financial difficulties aggravated by work leave (oftentimes informal work) leading to difficulties in nutrition, housing and transportation; and the absence of symptoms. In our culture, the association of a symptom with a disease is very striking. Therefore, if there are no symptoms, then the individual stops treatment because he feels cured. The knowledge about the disease that the patient needs must be increased by extending it to the community to ensure adherence and reduce the stigma; these factors were also suggested in the studies by Dias [21], Abebe [24] and Watkins [34]. Furthermore, the gender differences highlighted by Onifade [29] show that females suffer more pronounced adverse psychosocial and economic consequences compared with men.

The **medical obedience** category was discussed by the students in the focus group as a challenging situation with patients, especially male patients, with regard to non-adherence to prescriptions.

(…) *Usually*, *the man tends to disrespect more*, *I mean*, *not to disrespect more… to disobey the doctor more often…*

*(S—F)*

*It’s a more challenging stance*.

*(S—J)*

The issue of medical obedience noted by the students exemplifies a pattern of relationships in healthcare, especially in Brazil, in which patronizing care is predominant, attributed to the patients’ passive stance and lack of co-responsibility for treatment. Moraes [35] criticizes this stigmatizing stance and considers it a deviation of conduct by the patient. The future carers (students) in this study reproduced this way of thinking, disregarding the autonomy of the patient, his knowledge about the disease and the therapeutic repertories learnt and developed throughout life in the social and cultural context.

To face their illness and treatment, the patients needed social and emotional bonds, which were categorized as a **social network of support** (a group of interpersonal relationships perceived as significant). In the focus groups, a patient discussed the importance of support from a family member to treatment adherence:

*So if she doesn’t find support*, *not only from the multidisciplinary team*, *the doctor*, *psychologist*, *physical therapist*, *nurse*, *but especially from the family… that isn’t there*, *every day with her… giving strength*, *just like you had this disease and your wife was there by your side… definitely understanding this… then suddenly a huge fan opens*. *There are several possibilities*, *several factors that can interfere in this sense*.

*(P—15)*

Similarly, a student made the following comment:

*The family there*, *giving support*. *Encouraging… Saying it has to be treated because there is a cure… And that they are with him… the friends themselves… For the person*, *this improves*. *Even the immunity (…)*, *after the person… has the support of family*, *of animals*.

*(S—D)*

Reports from this study show that a good **bond with the healthcare staff**, opportunities for integrated care and a good **doctor-patient relationship** made it easier to maintain continuity of care and treatment adherence. Aiming to identify the effective barriers to TB control in Senegal, Hane [30] emphasized that a good doctor-patient relationship that stimulates dialogue must form the foundation of any strategy that aims to improve treatment adherence. According to Souza [31], the “meanings regarding the disease are built through the routine conviviality in the environment of health institutions”, which emphasizes that effective bonds with patients allows patients’ demands to be met and anticipated.

*… it’s a patient that we will follow for a long time and who needs to create a bond… to build trust (…)*. *If the patient doesn’t have trust*, *he won’t do it*. *He will at the beginning… but he’s not going to trust and will ditch the medication*. *We know that the patient has a lot of resistance to doing it*. *So I think the doctor has a more than fundamental role at this moment*.

*(Dr*. *2)*

In the discharge interview, a patient emphasized how much the bond with the healthcare professionals contributed to her treatment:

*I… I just didn’t really like getting sick*. *((laughs)) No one deserves this disease*, *but… about the treatment*, *I loved it*. *It was… the people… the doctors… you… everyone here*, *very caring… I even said today*, *outside*, *I said*, *“Look*, *the people here*, *we even miss seeing them… because they are very caring people… with us… with the medications… they worry… about me… I only have to praise*. *I told you*, *today*, *that they called me…*

*(P—16)*

The resources that the professionals offered the patients to facilitate adherence were categorized as **assistance strategies**, such as the ring of a bell in the ambulatory clinic if someone was discharged. This approach aimed to praise patients who were released and to encourage others to continue their treatments, in addition to establishing contact with patients who had missed their visit. Accordingly, the bonds among professionals, healthcare services and patients during treatment were highlighted, and their importance as a didactic and pedagogical resource must be emphasized in medical education.

*… when we entered the clinic*, *you notice that the doctor has a good relationship here*. *On that day*, *we stayed… He kept encouraging the patient to take the medication*. *We even saw in a consultation a patient that had just taken the medication… Then he rings the bell*, *he takes pictures… That whole thing*! *((laughs))*

*(S—O)*

*…Our adherence rate here in the hospital*, *because we are a specialized*, *tertiary hospital… is similar to the healthcare basic units*, *and we don’t have DOTS*. *If a patient was absent*, *we contact them*, *we call*, *send a telegram…*

*(Dr*. *7)*

The patients in this study used the following strategies to adhere to treatment: to mark the administration of medication every day on the calendar, to place reminders in the dresser where the medication was stored, and to control the number of medications. These strategies reveal the emotional investment of the patients, their personal commitment and interruptions in their routine. In another Brazilian study, the strategies suggested by patients were a decrease in medications and the time of treatment [36]. These proposals should be addressed in future TB control policies.

The doctors and students recognized that DOTS (*Directly Observed Treatment Short-Course)* positively influences adherence, although this practice is not used at this public university hospital. We included this practice in the **TB and society** category (situations involving health and TB policies in the community).

*…In many places what wins… A lot in terms of adherence is the DOTS strategy*, *where the patient has to go there every day or to the center*, *or the community health agent goes to the patient’s house… I think it doesn’t have to be for everyone*. *But as a public healthcare policy*, *this ends up showing results*.

*(Dr*. *7)*

In Kenya, Malawi and Uganda, the choice to use DOTS by patients has improved adherence rates [37]. However, DOTS can violate the autonomy of patients, their self-care and their right to privacy [38]. Hane [30] reinforced that DOTS must be adapted to local situations. The approach offered to patients must consider the complex range of issues involved in the experience of TB and its treatment.

The doctors and students mentioned that the patients used mental defense mechanisms to protect themselves from painful feelings; the **denial** category is presented in the literature as the mental defense most used by patients in the acute phase of diseases or in cases of serious prognosis [39]. This behavior was evident when the patients received a diagnosis that they did not believe or understand or when they did not hear anything the doctor had said after a consultation.

*I still can’t believe I have TB*. *I don’t feel anything*. *I don’t have any symptom that people talk about*.

*(P—1)*

It is important that the healthcare professional knows about the mental defense mechanisms most commonly used by patients to prevent negative impacts on adherence.

Another mental mechanism used was rationalization (pursuing a logical explanation to avoid one’s feelings during a significantly difficult experience). Rationalization is addressed in the literature as a defense mechanism that is very common among doctors [40]. In contrast to denial, the rationalization among patients in this study allowed them to comply with the medical therapy. If the patient was a healthcare professional, he or she felt a constant risk of contracting TB and perceived the workplace as threatening, fearing the recurrence of the disease after treatment completion.

*Because if it weren’t like this*, *I would have given up my profession already… I would look for something else to do*. *But no*! *((raises voice)) I was born to do this*! *If it means continuing the risk*, *I continue the risk*. *In regards to my health… I will keep risking myself here at the hospital*. *I was born to be a healthcare professional*. *I will keep having contact with people with tuberculosis*. *We don’t know who has tuberculosis*. *But if the person will depend on my assistance*, *I will be there attending… I don’t worry… I do my treatment*, *and I’m always attentive*. *This is the difference I always think about*, *you know*?

*(P—15)*

The psychosocial changes identified in the patient interviews showed aspects of the experience of TB and its treatment. These changes, containing negative meanings, challenge the patients, preceptor doctors and medical students to overcome these difficulties associated with TB.

### Training future doctors

Relevant factors related to medical teaching, care practices, perceptions and conceptions of the disease, and *actions and interactions* in this formative process were included in the **medical education** category.

The aspects verbalized by students pertaining to the assistance, to their learning and to the physical, emotional and social factors involving the patient in the treatment of TB were categorized as **difficulties of the student**. We highlight the difficulty of ascertaining patients’ understanding of the information supplied to them, the influencing factors emerging during treatment, a partial ability to learn the subjective aspects involved in the experience of falling ill, and the ability to empathize with patients.

The students in this study commented and lamented that despite the extensive course load, the medicine course was taught in a concentrated manner and prioritized theoretical activities, with little time remaining to experience the practice of ambulatory care activities. Many studies have also emphasized the harm of this extensive course load in influencing their learning and their health [41,42,43].

*The ideal would be for us to spend more time… To have followed those patients to see if that’s really true… If the adherence is really difficult*, *here in the ambulatory… If the adherence here is better… Maybe we would be more… In the know about this*. *About the situation of the patient with tuberculosis*.

*(S—O)*

Students also argued that theory and practice are very different, and the course should not be focused on books.

*In theory*, *everything is cute*, *right*. *(laughs)) In practice*, *it’s very different*. *Yeah… In theory*, *you think that the patient will have only that symptom… There each patient is a patient*, *not a book*. *I wish all the patients were like in the books*, *but…*

*(S—C)*

Two students said they did not know of the existence of extrapulmonary TB. This lack of knowledge was a concern of the preceptor doctor:

*And tuberculosis*, *they think it’s only in the lungs*. *My motto in tuberculosis*, *I say that to everyone*, *is… For the patient*, *for the student*, *for everyone… It’s tuberculosis*, *it can happen from the edge of the hair to the sole of the foot*. *So… Of course*, *the most common is the lung*, *but always think about tuberculosis*, *especially in our environment*. *Otherwise*, *we never… if we don’t think about tuberculosis as we should*, *we will never achieve the WHO goal*, *anyway*.

*(Dr*. *3)*

Although tuberculosis is an extremely prevalent disease in Brazil, the importance given to this disease in relation to other much less prevalent diseases is almost the same. The goal of the doctor was to extend the ability of the student to diagnose TB in our environment, where it is very prevalent. The goal is to decrease its incidence and to extend the knowledge that this disease can affect not only the lung but also several other organs. Most students became doctors without sufficient knowledge or experience in treating TB, one of the most prevalent diseases in Brazil. This situation may result in the misdiagnosis of TB and contribute to the long period of diagnosis and treatment for patients [44].

The conceptions of the fourth-year undergraduate medical students reflect the dynamics of their academic background, which emphasizes the biomedical model that focuses on somatic aspects rather than on a holistic and humanistic view. This factor is a major concern mentioned in a study by Koifman [41] regarding medical curriculum reformulation. Emphasizing the importance of integrated care in education and using it as a didactic resource in group tasks with patients would contribute to the feasibility of a more ethical and holistic approach to patient care.

The potential limitations of this study include the lack of a multi-professional team, as we focused only on doctors and students in one environment: the public university TB ambulatory clinic. This limitation may restrict comprehension of the concepts addressed. It was not possible to conduct a focus group with the doctors, and such a group would have yielded more data on these themes. The university hospitals are not representative of the Brazilian Unified Health System of attention and usually receive patients whose cases are more complex. However, this setting allowed us to interview medical students and doctors, which would not have been possible in the core network.

To better understand the experience of the illness and treatment of tuberculosis, this research offered an innovative way, within a single study, to approach the subjective aspects in intense, dynamic and procedural forms that emerge in clinical interactions among patients, doctors and medical students; these aspects are usually aspects in medical training. Therefore, the included subjects were directly involved in health care in an excellent context for learning: a public university hospital.

## Conclusion

This paper aimed to discuss the conceptions of medical students, doctors and patients within a single study and to evaluate the existing intersections in these conceptions relative to the knowledge and treatment of TB.

The stigma surrounding TB results in behaviors of self-segregation, concealment and mental defense mechanisms, such as denial in the patient. Patients also use rationalization to soften the feeling of vulnerability.

The patients reported experiencing the emotional repercussions of receiving the TB diagnosis. The doctors and medical students also experienced these effects when informing them and when identifying with the patients.

The bond with the healthcare staff, the doctor-patient relationship and health assistance strategies were concepts related to the facilitation of therapeutic adherence.

The following assistance strategies were reported as encouraging adherence: contact over the telephone with absent patients and the ringing of a bell by a patient when he or she is released from the clinic. These strategies also encourage other patients and exemplify, for students, the range of healthcare services.

The study showed the importance of the patient focus group in the second month of treatment and at treatment completion. The focus group was also an educational activity for students to elucidate experiences of the illness and treatment of TB patients and to emphasize the emotional, social and institutional repercussions involved.

The concerns regarding difficulties in assistance showed the need to prioritize education on diagnosing TB.

The treatment of TB is perceived by doctors and medical students as the responsibility of patients. The implications of medical obedience highlighted by the students signal the passive stance culturally attributed to patients.

## Supporting Information

S1 COREQ Checklist Consolidated criteria for reporting qualitative studies (COREQ) Checklist. (DOC)
